# Supplementary material for: End Sequence Analysis Toolkit (ESAT) expands the extractable information from single-cell RNA-seq data
Source: Genome Res. 2016 Oct;26(10):1397–410. doi: 10.1101/gr.207902.116 (PMC5052061; doi:10.1101/gr.207902.116)
Supplement: Supplemental Material [file supp_gr.207902.116_Supp_Materials.tar.gz › ESAT-esat_v0.1/doc/html/dir_11de5a41d3678d547170dda2eddc0468.html]

ESAT: java/broad/core/sequence Directory Reference


ESAT


- Main Page
- Related Pages
- Packages
- Classes
- Files

All Classes Namespaces Files Functions Variables Enumerator Pages

- java
- broad
- core
- sequence

sequence Directory Reference

Directory dependency graph for sequence:

|  |  |
| --- | --- |
| Files | |
| file | AbstractFastaParser.java |
|  | |
| file | Extractor.java |
|  | |
| file | FastaHandler.java |
|  | |
| file | FastaParser.java |
|  | |
| file | FastaQualityBinaryIO.java |
|  | |
| file | FastaQualityHandler.java |
|  | |
| file | FastaQualityIndex.java |
|  | |
| file | FastaQualityParser.java |
|  | |
| file | FastaSequenceIO.java |
|  | |
| file | Sequence.java |
|  | |
| file | SequenceRegion.java |
|  | |
| file | SequenceRegionWindow.java |
|  | |
| file | SequenceUtils.java |
|  | |
| file | TranscribedRegions.java |
|  | |
| file | TranscribedSequenceComposition.java |
|  | |
| file | WindowSlider.java |
|  | |


---

Generated on Fri Aug 15 2014 15:11:16 for ESAT by  

 1.8.7
